# Patterns and Severity of Hearing Loss in Patients Undergoing Pure Tone Audiometry in Eastern Nepal: An Observational Study

**DOI:** 10.31729/jnma.v63i290.9217

**Published:** 2025-09-01

**Authors:** Shivam Pandey, Shravya Singh Karki, Chahana Pathak, Kishor Aryal, Aman Pandey, Gopal Subedi, Sangita Bhandary

**Affiliations:** 1 B.P Koirala Institute of Health Sciences, Dharan, Nepal; 2 Department of Otorhinolaryngology and Head & Neck Surgey, B.P Koirala Institute of Health Sciences, Dharan, Nepal

**Keywords:** *audiometry*, *conductive hearing loss*, *hearing loss*, *pure tone audiometry*, *sensorineural hearing loss*

## Abstract

**Introduction:**

There is limited evidence describing the demographic patterns of hearing loss in the Nepali context. This study aimed to analyze the audiometrie profiles of patients undergoing pure tone audiometry in a tertiary care centre of Eastern Nepal.

**Methods:**

A retrospective analysis was conducted on all patients undergoing pure tone audiometry at B.P. Koirala Institute of Health Sciences, Nepal, between April 2023 and July 2024. Ethical approval was obtained from the Institutional Review Committee (IRC-163-081-82). Census sampling was used, and data on demographics and audiometric profiles were compiled in Microsoft Excel and analyzed with SPSS version 25.

**Results:**

Among 3,468 patients (mean age: 42 years, SD ± 20.27 years, M:F ratio 1:1.2), 2629 (75.8%) exhibited some degree of hearing loss. Mild hearing loss was observed in 1225 patients (35.32%). Sensorineural hearing loss was seen in 1010 (32.21%) male vs 1130 (29.77%) female ears and conductive hearing loss was seen in 476 (15.18%) male vs 620 (16.33%) female ears. Sensorineural hearing loss increased from 17 (6.59%) in children under 10 years to 165 (80.88%) in age 80 and above, while conductive hearing loss declined from 87 (33.72%) in those under 10 to 9 (4.41%) in above 80 years.

**Conclusions:**

In this cohort of participants, three-quarters exhibited some degree of hearing loss, with mild loss being more common. Sensorineural loss increased with age and predominant in males, while conductive loss was more frequent in younger patients and females.

## INTRODUCTION

Hearing loss ranks among the top three global causes of years lived with disability, affecting all age groups.^1^ By 2050, nearly 2.5 billion people are expected to experience some degree of hearing loss, with 700 million requiring rehabilitation.^2^ This impairment is strongly linked to reduced quality of life, including social difficulties and feelings of handicap.^3^ The prevalence and causes of hearing loss differ between high-income and developing countries.^4^ In resource-limited settings, preventable factors such as otitis media, impacted wax, childhood infections, noise exposure, and ototoxic drugs play a major role.^5^ Despite its global significance, hearing loss remains under-researched in low- and middle-income countries, including Nepal. Generating region-specific data is essential to guide interventions, inform health policies, and improve hearing care.

The objective of this study is to assess the severity, and types of hearing loss among patients undergoing pure tone audiometry at a tertiary care centre in Eastern Nepal.

## METHODS

This was a descriptive study conducted retrospectively at the Audiology Unit of the Department of Otorhinolaryngology and Head & Neck Surgery, B.P. Koirala Institute of Health Sciences (BPKIHS), Nepal. The study covered the period from April 2023 to July 2024.

Ethical clearance was obtained from the Institutional Review Committee (IRC-163-081-82) of the BPKIHS hospital prior to data collection. As this was a retrospective study using anonymized hospital records, individual patient consent was waived by the IRC.

All outpatients and inpatients who underwent their first Pure Tone Audiometry (PTA) during the study period were included. Patients were excluded if their PTA reports were incomplete (e.g., missing thresholds for any of the standard test frequencies: 250 Hz-8 kHz) or if demographic details such as age or sex were not recorded.

The final study population included 3,468 patients. Demographic details (age, sex) and audiometric thresholds were recorded. PTA was conducted using the Inventis Piano Plus audiometer (AU1CE22239781) in a soundproof environment. Air and bone conduction thresholds were measured across frequencies from 0.25 to 8 kHz. The pure-tone average (PTA) was calculated using thresholds at 0.5, 1, and 2 kHz. Hearing loss was classified by severity based on World Health Organization (WHO) criteria.^6^ For each ear with an air conduction threshold of less than 25 dB type of hearing loss was determined: sensorineural, conductive, or mixed.

Data were compiled and analyzed using Microsoft Excel and Statistical Package for the Social Sciences (SPSS) version 25 (IBM Corp., Armonk, NY, USA). Descriptive statistics were used to summarize demographic characteristics and hearing thresholds. Comparative analyses were performed based on age and gender to explore distribution patterns of hearing loss.

## RESULTS

The study sample population includes 3,468 people with a mean age of 42±20.27 years and median age 41 years (IQR: 25-57 years). Of these, 1,568 (45.21%) were male, 1,898 (54.72%) were female, and 2 (0.057%) individuals identified as “other,” resulting a male-to-female ratio of 1:1.2. The study population included 129 individuals (3.72%) under 10 years, 416 (12.00%) aged 10-19, 546 (15.74%) aged 20-29, 593 (17.10%) aged 30-39, 508 (14.65%) aged 40-49, 496 (14.30%) aged 50-59, 406 (11.71%) aged 60-69, 272 (7.84%) aged 70-79, and 102 (2.94%) aged 80 and older.

Among the participants, 839 (24.19%) had normal hearing. Mild hearing loss was observed in 1,225 (35.32%), while moderate or greater hearing loss was observed in 1,127 (32.49%) of participants. Unilateral hearing loss was seen in 277 patients (7.99%). Sensorineural hearing loss was observed in 2142 (30.88%) ear samples, conductive hearing loss was recorded in 1,095 (15.79%) and mixed hearing loss in 832 (12.00%) ears.

A gender-based analysis showed that females had an average hearing level of 37.66± 22.49 dB on right ear and 35.68±20.87 dB on left ear, while males had 38.87± 23.15 dB on right ear and 37.38±22.89 dB on left ear respectively.

**Figure 1:**
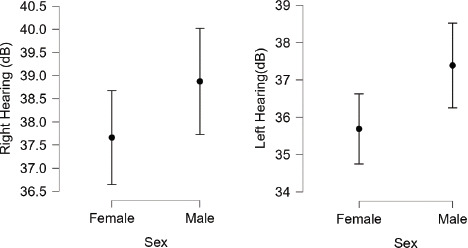
Interval plot of left and right hearing in male (n=1,568) and female participants (n=1,898).

**Figure 2:**
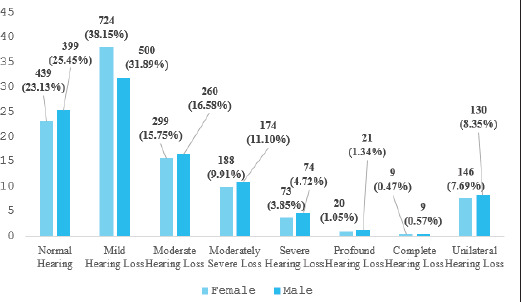
Bar graph showing the gender-based distribution of the severity of hearing loss (n = 3466).

On gender-based analysis, 439 (23.13%) female participants had normal hearing, 724 (38.15%) had mild hearing loss, 299 (15.75%) had moderate hearing loss, 188 (9.91%) had moderately severe hearing loss, 73 (3.85%) had severe hearing loss, 20 (1.05%) had profound hearing loss, 9 (0.47%) had complete hearing loss and 146 (7.69%) had unilateral hearing loss. For males, 399 participants (25.45%) had normal hearing, 500 (31.89%) had mild hearing loss, 260 (16.58%) had moderate hearing loss, 174 (11.10%) had moderately severe hearing loss, 74 (4.72%) had severe hearing loss, 21 (1.34%) had profound hearing loss, 9 (0.57%) had complete hearing loss and 130 (8.35%) had unilateral hearing loss.

**Figure 3:**
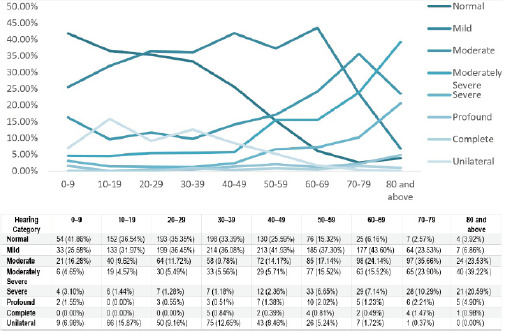
Age-based distribution of the severity of hearing loss (n = 3468).

An age-based analysis showed that normal hearing was observed in 54 (41.86%) participants aged 0-9 years and in 4 (3.92%) participants aged >80 years. There were 177 (43.60%) participants with mild hearing loss in the 60-69 years age group.

An ear-based analysis showed normal hearing in 1594 (42.0%) female ears and 1270 (40.5%) male ears. Sensorineural hearing loss was seen in 1010 (32.21%) males and 1130 (29.77%) females. Conductive hearing loss was seen in 476 (15.18%) male and 620 (16.33%) females.

**Figure 4:**
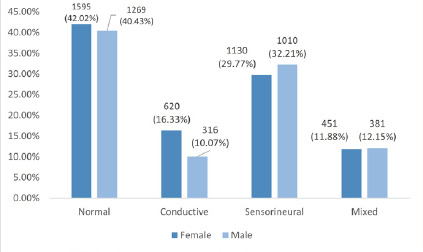
Bar graph of the gender-based distribution of the type of hearing loss (n = 6932).

An ear-based analysis of hearing loss types showed that a greater proportion of female ear samples fell within the normal hearing range compared to males (42.02% vs 40.47%). Additionally, sensorineural hearing loss was more commonly observed in males, while conductive hearing loss was more prevalent in females.

**Figure 5:**
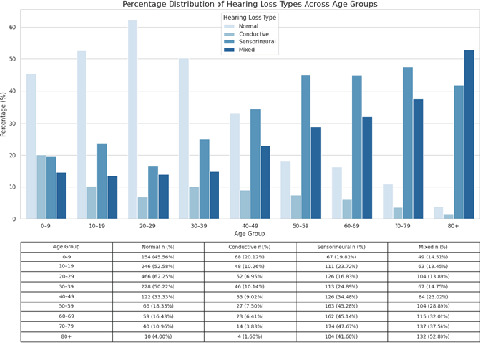
Bar diagram of the age-based distribution of the type of hearing loss (n = 6936).

Sensorineural hearing loss was present in 17 (6.59%) children under 10 years and 165 (80.88%) adults aged 80 and older, while conductive hearing loss was present in 87 (33.72%) children under 10 years and 9 (4.41%) adults above 80 years.

## DISCUSSION

In our study, 2629 out of 3,468 patients (75.81%) who underwent pure tone audiometry exhibited some degree of hearing loss. Mild hearing impairment constituted the largest proportion of cases, affecting nearly half of the individuals with hearing loss, followed by moderate hearing loss in approximately one-fifth and moderately severe hearing loss in about one-eighth of cases. Comparable findings were reported in a similar study by Khanal et al. at tertiary centre in Kathmandu, a regional study by Puthukudy et al. and both global and regional analyses conducted by Stevens et al. all of which demonstrated a similar trend of declining prevalence of more severe forms of hearing loss.^4,7,8^ The consistent pattern observed across these studies strengthens the validity of our findings and suggests that the distribution of hearing loss severity may be generalizable to clinical cohorts of patients with suspected hearing impairment, despite geographical and demographic differences.

In the present study, sensorineural hearing loss (SNHL) emerged as the most frequently observed type of hearing impairment, followed by conductive hearing loss (CHL) and mixed hearing loss, respectively. These findings are in concordance with those reported by Khanal et al. from a tertiary centre in Kathmandu, as well as regional studies conducted by Puthukudy et al. and Maiti et al., which similarly identified SNHL as the predominant pattern. In contrast, studies by Lageju et al., Dhungana et al. and Rabbani et al.^7-12^ reported CHL as the most common type. This divergence may be attributed to the demographic profile of their study populations, which included a larger proportion of younger individuals; an age group more commonly affected by CHL.

A gender-based analysis showed male predominance in hearing loss was across almost all severity levels. Also, the difference in hearing levels is consistent across both ears, with females demonstrating better hearing by approximately 1.21 dB in the right ear and 1.70 dB in the left ear. This is consistent with Khanal et al. study at Nepal Police Hospital, where 66% of those with hearing loss were male, and Puthukudy et al. research in India, which also showed a higher frequency of hearing loss in males (78.65% vs 77.67% in the right ear and 79.11% vs 77.86% in the left ear).^7,8^ Globally, hearing impairment is more common in adult males than females. Among those aged ≥15 years, 12.2% of males and 9.8% of females have hearing loss ≥35 dB. Males also have higher rates across all severities: mild (22.7% vs 19.0%), moderate (8.4% vs 6.8%), moderately severe (2.6% vs 2.0%), severe (0.8% vs 0.6%), and profound/complete (0.5% vs 0.3%).^13^ The higher male frequency of hearing loss may be linked to occupational and recreational noise exposure. Some researchers have proposed that the observed differences in hearing between males and females may be influenced by hormonal factors, particularly the protective effect of estrogen on auditory function.^14^ In contrast, Rabbani et al. in Bangladesh found a female predominance (58%) and Lageju et al. in Nepal reported a slight female predominance (51.3%).^10,12^ These differences may be due to variations in study populations or sampling bias due to small sample size.

Also, this study revealed a slightly higher frequency of sensorineural hearing loss (SNHL) among males, while conductive hearing loss (CHL) was more frequently observed in females. This pattern aligns with previous findings reported by Khanal et al. and Maiti et al. who attributed the higher burden of SNHL in males to greater occupational exposure to noise, lifestyle factors such as smoking and hormonal factors.^7,9^

Regarding the age-based analysis, our study revealed that the proportion of patients with normal hearing declined progressively from 41.86% in the 0 to 9-year age group to 3.92% in individuals aged 80 years or older. This trend aligns with the findings of Puthukudy et al. and Khanal et al. Additionally, our study demonstrated that conductive hearing loss was more frequently in younger age groups, while sensorineural hearing loss (SNHL) increased significantly with age, comprising 80% of cases in individuals aged 80 and above.^7,8^ This pattern mirrors the observations of Khanal et al., who noted a higher prevalence of conductive hearing loss in younger individuals and a progressive rise in SNHL among older age groups.^7^ The high prevalence of SNHL in the elderly population is likely attributable to presbycusis and cumulative exposure to occupational and environmental noise over time.

In a community-based survey by Maharjan et al., a prevalence of 5.73% for hearing impairment was reported among Nepalese school children, with conductive hearing loss being the most common type, affecting 70.47% of the cases.^15^ The study also identified several common causes of hearing loss in this population, including chronic otitis media, otitis media with effusion, and ear wax impaction.

Although the large sample size and diverse demographics of the participants strengthen the internal validity of the study and allow for cautious generalization to the clinical population with hearing impairment, several limitations must be acknowledged. Due to sampling bias inherent in hospital-based studies, these findings cannot be extrapolated to the general population, and the true incidence or prevalence of hearing loss at the community level cannot be accurately estimated. Additionally, the retrospective nature of the study restricted our ability to assess the etiological factors contributing to hearing loss, such as occupational exposure, chronic otitis media, or genetic predispositions. Other important limitations include the potential for inter-observer variability in audiometric testing, the absence of socioeconomic and educational data that may influence health-seeking behaviour, and the lack of longitudinal follow-up to assess progression of hearing loss over time. These factors should be addressed in future prospective, population-based studies to more comprehensively characterize hearing loss patterns and their determinants in Eastern Nepal.

Given the limited research on hearing impairment in Eastern Nepal, this study provides important insight into the prevalence, types, and severity of hearing loss in the region. Our findings align with both regional and international studies, underscoring the need for targeted screening programs and public health interventions. Future research should focus on prospectively identifying risk factors (such as occupational noise exposure and middle ear disease) and evaluating the effectiveness of hearing conservation strategies.

## CONCLUSIONS

In this cohort of 3,468 patients undergoing pure tone audiometry, approximately three-quarters exhibited some degree of hearing loss, with mild impairment representing the largest proportion. Sensorineural hearing loss occurred more frequently in males and increased with age, while conductive hearing loss was more common in younger patients and females. The proportion of individuals with normal hearing declined progressively across age groups. These findings highlight the patterns, types, and severity of hearing loss in a clinical population from Eastern Nepal.

## Data Availability

The data are available from the corresponding author upon reasonable request.
